# Key considerations when involving children in health intervention design: reflections on working in partnership with South Asian children in the UK on a tailored Management and Intervention for Asthma (MIA) study

**DOI:** 10.1186/s40900-022-00342-0

**Published:** 2022-02-28

**Authors:** Laura S. Nixon, Nicky Hudson, Lorraine Culley, Maya Lakhanpaul, Noelle Robertson, Mark R. D. Johnson, Melanie McFeeters, Narynder Johal, Charlotte Hamlyn-Williams, Yebeen Ysabelle Boo, Monica Lakhanpaul

**Affiliations:** 1 Population, Policy and Practice, UCL Great Ormond Street Institute of Child Health, London, UK; 2 School of Applied Social Sciences, De Montfort University, Leicester, UK; 3 Faculty of Biology, Medicine and Health, University of Manchester, Manchester, UK; 4 Department of Neuroscience Psychology and Behaviour, University of Leicester, Leicester, UK; 5 Mary Seacole Research Centre, De Montfort University, Leicester, UK; 6 NHS England, Direct Commissioning, Midlands Region, Leicestershire, UK; 7 Parent Representative, Leicester, UK; 8 Aceso Global Health Consultants Ltd., London, UK; 9 Nuffield Department of Population Health, University of Oxford, Oxford, UK; 10 Community Paediatrics, Whittington NHS Trust, London, UK; 11 Aquarius Population Health, London, UK

**Keywords:** Community health, Child Health, PPI, CBPR, Asthma, Intervention design, Paediatrics, Ethnic Groups, Diversity, Research Methods

## Abstract

Participatory research is an empowering process through which individuals can increase control over their lives, and allows researchers/clinicians to gain a clearer understanding of a child’s needs. However, involving children in participatory research is still relatively novel, despite national and international mandates to engage children in decision making. This paper draws on the learnings from designing the Management and Intervention for Asthma (MIA) study, which used a collaborative participatory method to develop an intervention-planning framework for South Asian children with asthma. There are currently 1 million children in the UK receiving treatment for asthma, making it one of the most prevalent chronic childhood illnesses. Symptoms of asthma are often underrecognized in children from South Asian communities in the UK, contributing to increased disease severity and increased attendance at the emergency department compared to White British children. Despite this, ethnic minorities are often excluded from research and thus absent from the ‘evidence base’, making it essential to hear their perspectives if health inequalities are to be successfully addressed. We worked alongside healthcare professionals, community facilitators, parents, and children to identify the key concerns and priorities they had and then designed the framework around their needs. Reflecting on the process, we identified several key considerations that need to be addressed when co-developing interventions with children. These include the power dynamics between the parent/researcher and child; navigating the consent/assent process; how parental involvement might affect the research; establishing a convenient time and location; how to keep children engaged throughout the process; tailoring activities to different levels of ability; and accounting for cultural differences. These factors were considered by the researchers when designing the study, however, implementing them was not without its challenges and highlighted the need for researchers to develop expertise in this field. Tailoring existing research methods allowed us to explore children’s perceptions, priorities, and experiences of illness more effectively. However, involving children in participatory research is a complex undertaking, and researchers need to ensure that they have the expertise, time, and resources necessary to be able to fully support the needs of child participants before deciding to commit to this approach.

**Supplementary Information:**

The online version contains supplementary material available at 10.1186/s40900-022-00342-0.

## Plain English summary

Involving families in health research gives children the opportunity to have more control over their healthcare and can help researchers/clinicians to better understand a child’s needs. However, involving children in research is still an uncommon practice despite international pressures to include children in decision making. This paper reflects on the development of the Management and Intervention for Asthma study, which worked with communities to develop a framework for planning health interventions for South Asian children with asthma. There are currently 1 million children in the UK being treated for asthma, making it one of the most common chronic childhood illnesses. Symptoms of asthma are overlooked in children from South Asian communities in the UK, leading to worse health outcomes and more visits to the emergency department. We worked with the community to identify the key concerns and priorities they had, and then jointly designed the framework around their needs. We identified several key things to consider when co-developing interventions with children:

- Power dynamics
- Consent/Assent
- Parental involvement
- Time and location
- Engagement
- Diversity of needs
- Ethnicity and Culture

Adapting our research and engagement methods to suit children’s needs enabled us to involve them in the study in a way that allowed us to effectively explore their views, priorities, and experiences of their illness. However, researchers need to make sure that they have the expertise, time, and resources to be able to support the needs of child participants.

**Supplementary Information:**

The online version contains supplementary material available at 10.1186/s40900-022-00342-0.

## Main text

Community-based Participatory Research (CBPR) has been increasingly embraced by the research community in recent years. CBPR actively involves community members in research to enhance the researchers’ understanding of the social and environmental factors impacting their area of study [1].

It not only provides researchers with richer and more comprehensive data but is also an empowering process through which participants can increase control over their lives [2]. Local expertise and knowledge of social structures can allow for the development of more effective interventions, since researchers can work alongside the community to establish a targeted method that would be most appropriate for the local population. Participatory research should be seen as being on a continuum from being minimally participatory to being fully egalitarian [3, 4]. CBPR, which involves the community co-developing the project alongside researchers, is on the other end of the spectrum to Patient and Public Involvement (PPI). PPI panels are another participatory method used to improve how studies are ‘prioritized, commissioned, undertaken, communicated and used’ [5], by consulting the public about their views and concerns. Despite the impetus from UK research councils to include participatory approaches in study design, a survey by INVOLVE showed that only 19% of respondents planned on including PPI in their study, and many who claimed that they would did not follow through with their plans [6].

Although children are increasingly included on PPI panels, their involvement in other forms of participatory research is still relatively novel. Despite the recent rise in popularity of CBPR, a systematic review of literature exploring youth community research found that only 15% involved the views of young people in their study [7]. Many were community-placed rather than community-based and did not actively engage the community in contributing to intervention or service design. This is despite the fact that the UN has been advocating for their involvement since the development of the UN Convention on the Rights of the Child (UNCRC) in 1989 [8]. Article 12 of the UNCRC asserts that children should have the right to be involved with decisions that affect them, not only in personal situations but in shaping the services they use [8]. However, it is important to ensure that they are not only involved, but that the methods used are ethically sound.

The Children’s Commissioner recently criticised the UK government for having an ‘institutional bias’ towards children, arguing that Members of Parliament do not engage with their young constituents and appear to be uninterested [9] in their wellbeing. Despite national policy mandates [10] to increase the weight given to the views and opinions of children and young people*,* the UK ranks poorly globally in providing an ‘enabling environment for child’s rights’ relative to the resources it has available [11]. This category prioritises the level of child participation in decision-making, the resources put towards improving children’s lives, and the level of discrimination between children from different backgrounds. Therefore, engaging with CYP needs to be made a priority for UK policymakers and research councils [12, 13], especially with regards to those from minority-ethnic backgrounds who are often underrepresented even further.

For example, in the UK minority-ethnic participants make up only 9.26% of participants in COVID-19 studies despite making up 13.8% of the population [14]. This figure is even lower for COVID-19 vaccine studies, with ethnic minorities making up just 5.72% of the participants despite being disproportionately affected by the virus [14]. Mixed and Indian ethnic groups in the UK are more than twice as likely to die from COVID-19 related complications; Pakistani, Bangladeshi and Black Caribbean populations nearly three times as likely; and Black African populations are more than four times as likely [15]. This is not specific to COVID-19; ethnic minorities tend to have worse health outcomes in general, including higher mortality rates for diabetes [16, 17]; cardiovascular disease [18, 19]; and higher maternal and infant mortality [20, 21].

Research suggests that one of the ways that these health inequalities can be reduced is by understanding the differing needs of these communities so that healthcare professionals can tailor their treatment and interventions accordingly [22, 23]. Therefore, it is essential that more is done to engage with communities to understand the factors that affect their healthcare so that interventions can be tailored to be effective as possible [24].

### The Management and Intervention for Asthma (MIA) study

The Management and Intervention for Asthma (MIA) study aimed to uphold these values by involving South Asian children diagnosed with asthma in designing the health intervention.

There are currently 1 million children in the UK receiving treatment for asthma, making it one of the most prevalent chronic childhood illnesses [25]. Symptoms of asthma are often underrecognized in children from South Asian communities in the UK, contributing to increased disease severity and increased attendance at the emergency department compared to White British children [26–28] despite presenting with lower frequency of symptoms suggestive of asthma [27]. This has been attributed in part to poor communication between clinicians and families, language barriers, and cultural/religious differences [29–31], yet there is little attempt to design interventions to address these challenges.

With this in mind, the MIA study aimed to develop an intervention-planning framework for South Asian children with asthma in the UK by using a tailored collaborative participatory method [32–35]. The study comprised of four iterative phases designed to explore the beliefs and perceptions of individuals, families, and communities: (1) a systematic evidence synthesis; (2) a community study; (3) a families and healthcare professionals’ study; (4) the development of potential collaborative intervention strategies [32]. These were used as part of a modified form of intervention mapping, i.e., a planning framework grounded in CBPR that describes an tailored systematic process for intervention development, implementation and evaluation [36]. Instead of relying solely on the reflections of their parents and practitioners, Phases 3 and 4 included engagement with children aged 5–12 years old with asthma (33 South Asian, 14 White British) by asking for their opinions on their healthcare needs. This gave them a direct voice that could be heard through the research and design of the intervention framework [37], and enabled the identification of key issues relating to the optimal management of asthma from children’s perspectives. Including the White British comparison group gave us a point of reference to identify culturally-specific issues [29, 38].

Research and engagement methods needed to be tailored to meaningfully involve young children, meet the aims of the study, and guide the outcomes [5, 13, 39]. Several approaches were used to encourage children to participate in the study and feel involved and heard during the research process. The children’s interviews explored their experiences of managing their asthma (Additional file 1: Annex 1: Topic Guide), while the prioritisation workshop saw children ranking the perceived importance of different aspects of their care [32, 34].

When planning the interviews and workshops, the following elements needed to be considered:

- Power dynamics
- Consent/Assent
- Parental involvement
- Time and location
- Engagement
- Diversity of needs
- Ethnicity and culture

### Power dynamics

Unequal power relations between children and adult researchers have been well documented in literature [40–42]. It is argued that a world created by adults leads to an inherent imbalance of power between adults and children [40, 42], which may be reflected in the research of children and young people. The autonomy of child participants can be threatened and lead to concerns over their freedom to participate, refuse, and withdraw from research or to give accurate views and experiences in a research setting [8, 42].

As such, negotiating the power dynamic between adults and children is one of the greatest challenges when developing interventions involving young people. If handled poorly, any information collected could be subject to bias and reflect what the children believe the researcher wants, rather than a true representation of their own thoughts. Children are prone to giving answers that they believe the adult wants to hear, which can be further exaggerated in a formal research setting [43, 44]. To minimise any pressure to conform, the interviewer on our project was a clinical research fellow (DB) who had experience working with children as a paediatrician.

However, child–adult power relations are more complicated than the dichotomy of ‘power’ or ‘no power’ [45]. Power exists on a spectrum, and simplifying the dynamic ignores social, economic, and cultural context [46]. In the case of our study, the power imbalance was heightened by the fact that our participants were from a minority-ethnic background. Being a part of a minority group often means receiving unequal treatment and having less power in society [47]. Interviewers need to be aware that this dynamic exists and be sensitive to this power imbalance so that they can remain as unbiased as possible and make families feel more comfortable. It is important to note that even if researchers make efforts to reduce risk of bias, interactions are unlikely to be entirely value-free [48]. Ethnic minorities may perceive stigma based on previous experience, so a difference in researcher/participant ethnicities could still influence the interaction [49]. Children may arguably be less aware of this divide, but research shows that children as young as five have downplayed their own South Asian identities in favour of whiteness [50], and will also be sensitive to their parent’s non-verbal cues.

Reflexivity on the part of the researcher is crucial, requiring them to continuously scrutinise the situation to ensure that the child is comfortable, understands the task, and does not feel pressured [51, 52]. In our case, reflexivity included paying attention to non-verbal cues about concerns over children’s experience of asthma management. For example, when a child suddenly became quiet and less responsive when asked about their experience of asthma management at school, the interviewer had to proceed sensitively and deftly to change the line of questioning. It is ethically essential to use this power advantage to ensure the comfort of the participant rather than aiming to achieve co-operation or simple compliance.

### Consent

As participants under the age of 16 cannot legally consent, there is often discussion surrounding the ethics of including children in research [53]. British medical law determines whether a minor has a right to consent to medical treatment by deciding if they are ‘Gillick Competent’ – that is, whether they can understand the choice they are making, weigh up the risk and benefits, and consider the longer-term impacts of the decision [54]. However, this does not legally follow in terms of research, meaning that children can only confirm their willingness to participate through ‘assenting’ rather than ‘consenting’. Despite assent not being a legal requirement, confirming that a child fully understands to what they are agreeing meets our ethical duty to ensure the child’s wellbeing. This is a concern that must be weighed up against the ethics of not permitting children a say in their own healthcare. After deliberation, we established that informed consent would be taken from the parents and children aged 10–12 who were deemed to be Gillick Competent [54] by our Paediatrician/Clinical Research Fellow, while younger children would give verbal assent*.* The study was explained thoroughly to the parents first, and once they had consented the children were asked to confirm their willingness to take part after an appropriate explanation of the activities. As laid out in the NSPCC’s research guidance, “the child’s wishes should be paramount”, therefore we established that if a child did not consent or assent to participate it overrided the consent from the parent/guardian [55]. Information was available in multiple South Asian languages on request to ensure that second-language families felt that they thoroughly understood the process. Consent should be an ongoing process [56, 57], so the team ensured that every child involved in the study knew that they were able to leave at any point.

Furthermore, parents or legal guardians acting as ‘gatekeepers’ may influence a child’s decision to participate in research [43]. This may mean that children are either coerced into, or denied participation in, a research project. Children may also lose interest during the activities but continue because they feel like they cannot exercise choice. During our study, a researcher noticed that two of the children showed a lack of interest in being interviewed and so, after discussion, withdrew them from the study. The children did not explicitly state their discomfort, nor did the parents, however the researcher’s probing revealed that the children would rather not be involved further. It is often difficult to ascertain the difference between disinterest and a child simply not conforming to the expectations of an interview setting. Children will often misbehave or derail an interview to assert control over a situation while still being content with participating, or may prefer to express themselves in a different way [45, 58]. The sensitivity with which young participants were treated in this study resulted in rich data, which highlights the need for researchers to have training and experience working with children if they want to engage meaningfully.

However, as the MIA study was underpinned by participatory research principles, parents and guardians were closely involved in the project’s shaping, design, and outcomes from its outset. Some parents and guardians were closely involved with the project as key stakeholders, giving them a vested interest in their children’s participation in the study. We attempted to mitigate this issue by recruiting interviewers with the experience, competence, and sensitivity to address any non-verbal cues indicating a child’s discomfort.

This reflexivity extends beyond assessing whether someone wishes to leave the study, to knowing when to adapt questioning in response to a child’s discomfort or distress. Situations such as this raise an important point regarding the difference between meeting the legal and regulatory research requirements and truly considering the wellbeing of each of your participants. We discouraged children from participating without parental agreement, but researchers need to consider and plan for a child wishing to participate despite parental opposition or without their knowledge. We believe that by continuously assessing children’s willingness to participate we met our ethical responsibility to ensure the participant’s wellbeing.

Despite consent and assent being challenging to negotiate when working with children, reflexivity employed by experienced researchers can help to overcome these barriers through safeguarding the emotional security of the participants [59].

### Parental involvement

The concerns regarding consent and power relations raise an important question of the extent to which parents should be involved in the research process. The balance between consent, quality of research, child comfort, and the role of the parent is a concern that must be handled delicately. However, there are currently no definitive guidelines on how to effectively involve parents in child-centred research [60]. In our experience, we found that while parental presence can be reassuring to young children, it can also be detrimental to the study. For example, during Phase 3, parents were initially invited to introduce themselves at the beginning of the interview. This enabled children to feel comfortable and at ease when speaking to the adult researcher. However, parents frequently introduced the interviewer to the child, for example, as ‘a doctor who wants to ask you questions about your asthma’, thus, framing the tone of the interview and immediately defining the power dynamics between the interviewer and the child. These first impressions are hard to negate.

We aimed to enable the children to speak to our researchers in private, however, we noted a number of dynamics that needed to be taken into account. Some parents found it difficult to leave an interview once it had commenced, and their presence may have acted as a ‘gatekeeper’; this may have potentially influenced children’s responses, magnifying the social desirability of their answers and changing the dynamics of the interview from a child-only interview to a family interview. This is an issue often faced by systemic/family therapists, as there is a natural tendency to talk *about* the child when other family members are present, rather than *with* the child as intended [61].

During Phase 4 this was mitigated by conducting the parents’ focus groups at the same time as the children’s prioritisation workshop [32, 35]. By conducting both sessions in the same hall simultaneously, we allowed for both sides to answer free from judgement whilst minimising the stress of being separated from each other. For future studies, a clear protocol describing the interview setting with strict restrictions on parental/guardian presence may help to reduce this effect. However, the benefits of parents/guardians being present should not be ignored, as the child may feel more relaxed and therefore more likely to open up.

Overall, parents are an inextricable part of child-centred studies that need to be managed carefully to ensure that their role is neither detrimental to the comfort of the child nor the quality of the data. More research into their influence on child-centred participatory research is needed.

### Time and location

Research context and setting can also play an important role in influencing the power imbalance when interviewing children. A child who is in a familiar environment such as their own home is more likely to feel relaxed during the interview [41]. Research is an unknown concept to many children, making it vital that they feel in control of their situation.

An unavoidable challenge that we faced was that parents were the ones who determined the time and venue of the interviews. Parental choice of time and venue does not take into account what the child would feel more comfortable with, potentially creating a conflict between the needs of the parent and child. Engaging with the needs of parents is an essential part of recruitment, however, discussing the child’s requirements when organising the appointment may increase the comfort of the child and the quality of the research. To create more flexibility for the families, childcare was provided onsite for those who had additional younger children. This made finding time to participate less stressful on the parents, which gave them more opportunity to better suit the appointment to their child’s needs.

When planning the time of the interviews and workshops, we also had to consider the fact that many South Asian families visit their country of origin over the summer holidays, and that some may have had religious commitments on certain days of the week.

When deciding on location, we considered conducting the interviews in schools, however, research carried out in schools (an environment connoting authority) can make a child more inclined to answer ‘correctly’ to interview questions [41, 62]. Therefore, we decided to conduct the interviews in children’s homes which also minimised the amount that participants had to travel to other venues. If working with low-income families, having to travel could be a barrier to participation due to the cost of petrol or public transport. This means that when choosing a location for workshops/interviews, the venue should ideally be accessible to the target population (e.g. walking distance, close to transport links). If this is not possible, then researchers could consider funding the participants’ travel costs.

However, despite the benefits, it is also worth noting that a home setting may not be appropriate for researching more sensitive topics (e.g., sexual health or family relationships) in case personal information is overheard by family members/housemates.

### Engagement

Developing ways of keeping children engaged and focused during long sessions is often overlooked in research settings. To work effectively with children, research methods need to engage the children’s interest and account for variations in age, understanding, and ability [63]. This influenced our decision to use discursive workshops and visually-oriented methods rather than more didactic and written methods typically used with adults.

We involved the children in Phases 3 and 4 of the study: the qualitative interviews and the co-development and prioritisation workshops. We used alternative participatory methods of data collection such as drawing, games, and youth facilitators in addition to the traditional interview [64, 65]. The first activity had the children prioritise key themes via diamond or linear ranking, known to be successful with children [66, 67]. The second was a budget pie game, where each child could distribute a virtual wallet of £300 across different priorities [35] to get a more nuanced understanding of the child’s primary concerns. Providing flexibility and a choice of tasks was also important and splitting the session into multiple activities made the process more interactive and engaging.

One approach we adopted during the qualitative interviews was to encourage children to express themselves by drawing pictures. The decision to use this technique was based on published reports that have proposed that using task-based activities as part of semi-structured interviews allows researchers to dive deeper into children’s ideas and concerns [64, 65, 68]. It has been suggested that activities such as drawing and writing can help to reduce the effect of unequal power relations, as these activities do not put children under pressure to provide the ‘right’ answer [41, 58].

Following the involvement of children in the qualitative interviews, they were invited to join the co-design and prioritisation workshop. To ensure inclusion of children and young people in this phase of the study, the research team recruited teenage youth facilitators (aged 15–16 years) through local networks to feedback on the workshop design and support the workshop. Before the activities, the research team ensured that the young researchers understood the aim of the project and trained them how to facilitate the research and work with the children. They were supervised throughout the workshop. One of the youth facilitators themselves had been diagnosed with asthma and, as an expert by experience, was able to share her personal reflections on the study questions and findings. It can also be noted that one youth facilitator was from a South Asian background. We felt it was important to have at least one facilitator be of a similar ethnicity to the children, as it is known to help establish trust between participants and the research team; this has also been shown to increase study retention rates [69].

The youth facilitators’ role included receiving the children and young people at the workshop, The youth facilitators were closer in age to the children than other members of the research team. It was felt that having someone in the workshop who was closer in age would put the children more at ease, thereby improving engagement in the workshop activities. The children’s workshop was held at the same time as the parents’ workshop but in either a separate room or on a stage behind a curtain. The youth facilitators were always paired with a researcher to ensure the safety of both the children and the youth facilitators. We noted that our approach significantly reduced the communication barriers between the research team and the children.

Tailoring the experience to keep children engaged did not come without its challenges. The activities increased the length of time required for the workshops, which placed an additional burden on the child and researcher [52]. Furthermore, despite the work put in to make the experience more engaging, some children who had been involved in the qualitative interviews still did not wish to participate in the workshop activities provided. Consideration should be given as to whether multiple shorter activities prior to the main workshop may be helpful to enable the child to develop familiarity with the researcher and youth facilitators, enabling them to build stronger personal relationships and prevent potential loss of interest during the activities.

### Diversity of needs

It is easy to make the mistake of assuming all children will respond to the same approach, rather than recognising that different groups may have different needs or interests. For example, our participants’ ages varied from 5 to 12 years old. As such, it was important to tailor the intervention not just towards ‘children’, but towards the specific demographics and level of socio-cognitive development.

The prioritisation activity was adjusted for different age groups by allowing older children to choose between linear ranking or a modified diamond ranking, while younger children exclusively used modified diamond ranking [35, 67]. This decision was taken as being able to position answers equally can make it easier for children to complete prioritisation tasks [70, 71]. Answers were also written on cue cards for the children to physically place in order, which simplified the activity [72] as it made it visual and reduced the need for abstract thinking.

If studies are limited to an interview setting, the key focus should be tailoring questions to meet the child’s ability level. Open-ended questions are recommended in qualitative research [73, 74] but are more difficult to adopt when interviewing young children. During this study, children struggled with answering hypothetical questions and responded more easily to closed questions, which limited the depth of the interviews. This experience was in line with Irwin and Johnson [75] and Wilson and Powell [62], who reported that closed questions were less taxing on a child’s linguistic and reasoning skills and were deemed less daunting. In a similar vein, the British Police are advised that when interviewing children their questions should be ‘simple, contain only one point per question, not contain abstract words or double negatives, and lack suggestion and jargon’ [76]. However, studies on affirmation bias conclude that young children are more likely to answer yes to a ‘yes or no’ question [73], so simplicity may not always be the best solution.

Another factor to consider was that some of the children involved had endured severe asthma attacks. Recalling these events can be traumatic, so phrasing questions in a way that would not trigger a negative response was essential [74]. This was mitigated by using an interviewer who had experience with children, by using open-ended questions, and by monitoring the child’s body language [74]. It is important to note that when designing a study addressing a sensitive topic, researchers must conduct a risk–benefit analysis to decide whether the risk to the child’s wellbeing makes the study unfeasible [12, 52]. Furthermore, drawings helped us to overcome some of the language barriers that restricted the child’s ability to articulate the more complex emotions related to their experience of asthma.

### Ethnicity

A consideration of ethnicity was central to all aspects of the research process, including the research design, methodology, and data analysis. This was achieved by involving specially-trained community facilitators who played a key role in the design, recruitment, and data interpretation [35]. Parent representatives and the community facilitators were also paid members of the research advisory group.

Trust is a considerable barrier to participation within minority-ethnic communities [77]. As such, we used community facilitators and local religious organisations to aid our recruitment. Personal engagement can assuage the potential fear that participants would be ‘guinea pigs’, or that they would be enhancing academic careers with little benefit to the communities concerned [69].

Researchers must adopt a stance of ‘cultural humility’, that is, self-evaluating and self-critiquing to maintain mutually respectful dynamic partnerships [78]. Cultural humility and critical reflexivity is essential at every stage of the research process [79]. The research team were very mindful of the need to reflect on the methodological approach best suited to the intended participants. This was aided by the close involvement of community members in the research design and implementation [35].

All the children in the study spoke English, however several of the parents were not fluent English speakers. In this case, the community facilitators were available to take consent in the parents’ preferred language. This step was taken to ensure that consent was valid and to avoid marginalising non-English speaking parents who wanted to be involved.

Another aspect of the study that was tailored to the South Asian community was the food that was offered to participants after the workshop. The catering was primarily food of South Asian origin and included vegetarian and halal options. This was important in maintaining a mutually respectful relationship with the community involved and to support local community-based facilities and providers [78]. As such, several of the initial focus groups for the study and the final intervention-design workshop were held in local minority-ethnic community centres.

## Conclusion

Developing the MIA intervention framework in partnership with children with asthma was an informative process, allowing us to learn from their lived experiences with asthma while developing further understanding of how best to negotiate involving CYP in CBPR. The study allowed researchers to build a positive relationship with the community, gain an awareness of children’s understandings of their asthma, and engage with their concerns. Although our study was conducted in 2018, before COVID-19 presented, the pandemic has undoubtedly presented new challenges and barriers for researchers. The remote conduct of research via online platforms raises uncertainty, notably not knowing: if a child has sufficient privacy to talk freely; how to engage children online; and how to build a relationship with a child while wearing a mask or over a video call. Even when engaging in person, using masks may make it difficult to connect with a child. These are concerns that need to be explored further, but the fact remains that the child’s safety is always the primary concern [80].

We have argued that expertise and consideration are needed to design and enact a study tailored to the needs of children, and that effectively respecting ethnic diversity requires a highly reflective and inclusive approach. It is imperative that the implications of diversity are considered fully from the outset; that the research team includes members of the communities concerned; and that the team questions at each stage how best to engage with differences of age, gender, ethnicity, and marginalisation. Such research may also incur additional costs, for example, using translated materials, involving and training community members to be research partners, and assisting participants with travel. This needs to be acknowledged by research funders, but much can be achieved with a research team who takes diversity seriously and continuously considers how the research and interventions can best meet the needs of those it is intended to reach.

We have presented a clear rationale in this paper for the ethical and essential inclusion of children in research and as part of the co-development of interventions that directly affect them. Age and ethnicity need to be prime considerations in research design and implementation, and our reflections on the MIA study provide guidance on how to tailor studies effectively.

These principles need to be acknowledged by the wider paediatric research community if we are to provide the best possible care for our children.

## Supplementary Information

**Additional file 1.** Semi-structured Interview Guide.

## Data Availability

Not applicable.

## Abbreviations

CBPR: Community Based Participatory Research
PPI: Patient and Public Involvement
MIA: Management and Intervention for Asthma study
CYP: Children and Young People
UNCRC: United Nations Convention on the Rights of the Child
